# Characterizing safety, toxicity, and breast cancer risk reduction using a long-term fulvestrant eluting implant

**DOI:** 10.1038/s41598-024-77186-z

**Published:** 2025-01-24

**Authors:** Scott Thomas, Elysia Roche, Pujan Desai, Nela Pawlowska, Diana Bauer, David Gingrich, Emily Hsu, Amelia N. Deitchman, Fran Aweeka, Pamela N. Munster

**Affiliations:** 1 Division of Hematology and Oncology, University of California, 1450 3rd Street, San Francisco, CA 94143 USA; 2 Laboratory Animal Resource Center, University of California, San Francisco, USA; 3 Drug Research Unit, Department of Clinical Pharmacy, University of California, San Francisco, USA

**Keywords:** Breast cancer, Drug development

## Abstract

For individuals at high risk of developing breast cancer, interventions to mitigate this risk include surgical removal of their breasts and ovaries or five years treatment with the anti-estrogen tamoxifen or aromatase inhibitors. We hypothesized that a silicone based anti-estrogen-eluting implant placed within the breast would provide the risk reduction benefit of hormonal therapy, but without the adverse effects that limit compliance. To this end, we demonstrate that when placed adjacent to mammary tissue in the 7,12-dimethylbenz[a]anthracene-induced rat breast cancer model a fulvestrant-eluting implant delays breast cancer with minimal systemic exposure. Using adult female sheep, surgical placement of fulvestrant-eluting implants was safe and did not elicit significant breast tissue pathology when placed at the base of the udder for directed elution into the mammary tissue. At 30 days of elution, fulvestrant was found to penetrate mammary tissue forming a concentration gradient beyond 15 mm from the implant. Consistent with the small animal rat study, minimal systemic fulvestrant biodistribution was found. Together, these studies provide the proof of principle that a breast indwelling fulvestrant-eluting implant can reduce the risk of breast cancer and limit systemic exposure, while penetrating and distributing through breast tissue.

## Introduction

In the United States, over 297,000 women are estimated to be diagnosed with breast cancer in 2023 and more than 43,000 will succumb to it^1^. Breast cancer prevention strategies include risk-reducing mastectomies or tamoxifen for premenopausal women, or aromatase inhibitors. However, the uncertain benefits for the individual and the impact of side effects render both surgical and chemical interventions undesirable for most^2–10^. The increasing number of patients at high risk identified by the recent surge in genetic testing, has rekindled the interest in developing novel disease prevention strategies.

An estimated 5–10% of breast cancers are linked to hereditary mutations, most commonly within the BRCA1 or 2 loci, which increases lifetime risk as high as 60–85%^11–14^. BRCA mutation carriers are also at higher risk for developing secondary breast cancers after initial diagnosis in either the same or contralateral breast^15,16^. The affordability of germ line testing and increased awareness has led to a substantial increase in women getting tested who now present with a definable breast cancer risk. Many women are, hence, aware of their elevated risk when still in their 20’s and 30’s, thus necessitating changes in current screening and prevention strategies.

Approved breast cancer prevention options include bilateral mastectomy, or systemic treatment with an anti-estrogen such as tamoxifen, or the aromatase inhibitors exemestane or anastrozole for postmenopausal women. For high-risk patients, bilateral mastectomy with or without accompanying oophorectomy reduces the risk of breast cancer by more than 95%^17–19^. Although effective at reducing risk, this option is highly invasive and irreversible, with tremendous subsequent mental and physical anguish. The alternative is 5 to 10 years of systemic anti-estrogen treatment. To date, tamoxifen has been the only approved drug for premenopausal women over 35. Despite a 50% relative-risk reduction in (*e.g.*high-risk patients defined with > 1.7% 5-year cumulative risk or history of LCIS) reported in a large, randomized trial of over 10,000 patients, the acceptance and adherence rate to the full course of tamoxifen is low. Undesirable side effects associated with systemic exposure and the concerns of long-term sequelae, such as stroke and endometrial cancer, have further limited interest^20–23^. In addition to BRCA2, recent studies show that mutations in PALB2, ATM, CHEK2, and TP53 genes, and to a lesser extent BARD1, RAD51C and RAD51D, significantly elevate lifetime breast cancer risk^24,25^. Consistent with its correlation with triple negative breast cancer, benefit to patients with BRCA1 mutations is not clear^26^. Furthermore, a strong family history of breast cancer may compound the risks in known and unknown low penetrance genes^27^. Nonetheless, risk reducing surgeries are not typically advised for carriers of such mutations.

To circumvent the side effects associated with systemic anti-estrogen therapy breast cancer risk reduction, novel localized delivery approaches have been explored. One such approach is local transdermal therapy, whereby estrogen receptor (*e.g.* 4-hydroxytamoxifen and endoxifen) or progesterone receptor (*e.g.*telapristone acetate) modulators are formulated into a hydro-alcoholic gel and applied directly to breast skin^28^. Both transdermal 4-hydroxytamoxifen and telapristone acetate have been evaluated in neo-adjuvant phase II studies enrolling patients with estrogen receptor positive ductal carcinoma in situ (DCIS)^29^or in patients with or without localized breast cancer scheduled for mastectomy^30^, respectively, and randomized against patients receiving comparable systemic therapy. These studies demonstrate the respective therapeutic is delivered across the dermal barrier and into underlying adipose/glandular tissue, but with varying success. Transdermal delivery of 4-hydroxytamoxifen decreased Ki-67 expression, the primary study endpoint, in DCIS lesions and resulted in tumor adjacent mammary tissue drug levels comparable to patients receiving oral therapy (5.8 versus 5.4 ng/g, respectively). Furthermore, systemic exposure was reduced roughly fivefold with the transdermal application. However, in a randomized double-blinded phase two study comparing oral tamoxifen (20 mg/day) to transdermal 4-hydroxytamoxifen (2 mg/day per breast) in patients (N = 75) prior to surgical resection of DCIS, transdermal delivery was not found to be non-inferior to oral tamoxifen^31^. Similarly, telapristone acetate breast tissue levels achieved from a transdermal application were significantly less than those in patients receiving the drug orally, thus, illustrating the challenge and differential transdermal delivery of small molecule therapeutics. The need for continuous daily self-administration of these transdermal formulations increases the risk of non-compliance.

Alternatively, our group has sought to deliver breast cancer risk reducing therapy locally via an indwelling drug eluting implant. Modeled after other long-term implants used for contraception, such as the levonorgestrel-releasing intrauterine device Mirena™, we tested the hypothesis that silicone tubing could be used to depot the anti-estrogen fulvestrant and facilitate its controlled release. Fulvestrant was chosen as the active agent to be delivered due to its proven efficacy in patients, relative potency against the estrogen receptor, high lipophilicity, not requiring metabolism, unlike tamoxifen, to be fully active, as well as its long-established safety history^32^. This proof of principle study demonstrated that when sealed within the lumen of silicone tubing, fulvestrant exhibited zero order elution by passive diffusion for an estimated duration exceeding five years, remaining stable and active^33^. In vivo murine experiments showed eluted fulvestrant maintained preferential on target (*e.g.* mammary tissue) versus off target systemic levels (*e.g.* major organs and plasma) and inhibited breast cancer xenograft growth and molecular targets comparable to systemic treatment.

Our initial study supported locally delivered fulvestrant as a means to treat breast cancer, while minimizing systemic exposure. However, the most likely application of this approach is in the prevention setting and the potential of this approach to prevent or reduce the risk of breast cancer developing remained unclear. Thus, in the current study, we extend these findings by demonstrating that a long term fulvestrant eluting implant directly placed into mammary tissue reduces the risk of breast cancer by delaying tumorigenesis in a rat breast cancer model. We further show that a scaled fulvestrant eluting implant can be placed within mammary tissue of female sheep without significant adverse effects and that eluted fulvestrant passively diffuses and penetrates through the glandular tissue.

## Materials and methods

This study was reported in accordance with ARRIVE guidelines.

### Implant fabrication and fulvestrant extraction

Fulvestrant was purchased from Toronto Research Chemicals (Toronto, ON). Silastic MDX-4210 medical grade elastomer and curing agent were purchased from Dow Corning (Auburn, MI). HelixMark platinum-cured silicone tubing was purchased from VWR (Radnor, PA). Fulvestrant eluting implants were made by hand mixing MDX-4210 elastomer and curing agent (9:1 parts, respectively), to which fulvestrant stock (150 mg/mL in EtOH) was added (25 mg/g) and mixed. Using a syringe, HelixMark silicone tubing (1.96 mm outer diameter, 1.47 mm inner diameter) was filled with the fulvestrant-elastomer mix and allowed to fully cure at 70 °C overnight. For the rat study, cured implants were cut to 4 cm lengths for subsequent use. For sheep studies, 50 cm of cured implant was arrayed in a spiral pattern and over-molded with MDX-4210 elastomer silicone to form a round base (6 cm diameter, 0.3 cm thick) such that half of the spiraled implant was embedded in the base and half protruded. Fabricated implants were sterilized by ethylene oxide incubation for 12 h, followed by off gassing for 24 h. To extract fulvestrant, implants were cut to 1 cm lengths and incubated in tetrahydrofuran for 20 min while rocking at room temperature. Acetonitrile and water were added to a final ratio of 10% tetrahydrofuran/ 45% acetonitrile/ 45% water and vortexed. Extracted fulvestrant was then quantified by high pressure liquid chromatography (HPLC).

### In vitro elution studies

Cured 4 cm length fulvestrant implants (N = 5) were individually transferred to vials containing 1.8 mL 1% sodium dodecyl sulfate (SDS) and incubated for the indicated times at 37 °C while rocking. After each time point, implants were transferred to new vials containing 1.8 mL 1% SDS and incubated at 37 °C. Implants were allowed to elute fulvestrant for a total of 194 continuous days. For samples continuously collected during this period, fulvestrant was subsequently quantified by HPLC.

### HPLC quantification

Fulvestrant eluted in in vitro studies or from implant extraction was quantified using an Agilent (Santa Clara, CA) 1100 series HPLC coupled to a 10 cm Agilent Eclipse Plus C_18_ column. Injected samples (10 µL) were separated using an isocratic method employing 95% acetonitrile in water with a flow rate of 0.3 mL/minute. Eluted fulvestrant was detected at 210 nM using a diode array detector with a retention time of ~ 5.1 min. To quantify, fulvestrant standards (2.5–40 µg/mL) were prepared in appropriate matrix, which resulted in a lower limit of quantitation ~ 2.5–3 µg/mL. Peak area under the curve was determined using ChemStation (Agilent) and standard linear curve fit and fulvestrant quantification were determined using GraphPad Prism version 10.0.2.

### Rat breast cancer prevention study

Female Sprague–Dawley rats (N = 90) (Charles River Labs), 175-215 g, were randomized into three cohorts: (1) No drug (N = 23), (2) systemic fulvestrant (N = 29), and local fulvestrant (N = 28). The study was designed by the UCSF Biostatistical Core such that N = 25 rats/cohort were required to achieve a greater than 80% power. Based on previous fatality rates following 7,12-dimethylbenz[a]anthracene (DMBA) treatment, additional animals were included in the study to account for loss prior to study initiation (Day 1 dosing). As such, animal fatalities prior to dosing were excluded from statistical comparisons: N = 7 from no drug, N = 1 from systemic fulvestrant, and N = 2 from local fulvestrant cohorts. The study was not conducted blindly. Rats in the locally treated cohort received 4 cm fulvestrant eluting implants (N = 4 per rat) placed subcutaneously running cranial to caudal under the teats and adjacent to mammary tissue. Two were placed roughly the length of the thorax spanning the upper two quadrants, left and right side, and two were placed across the abdominal and inguinal region spanning the lower two quadrants, left and right side. Rats in the no drug cohort received sham implants not containing fulvestrant, comparable in surgical placement to the local fulvestrant cohort. Concurrently, rats in the systemic cohort were injected subcutaneously and dorsally between the shoulder blades with 0.1 mL 12.5 mg/kg fulvestrant in peanut oil, and weekly thereafter. All animals received a single dose of 7,12-dimethylbenz[a]anthracene (20 mg in 1 mL peanut oil) by oral gavage, two weeks following surgical implantation or oral drug dosing. The mammary region of animals was manually palpated weekly until the first tumor was detected in the first animal. Subsequently, all animals were evaluated, and tumor volumes (width^2 ^× length/2) were measured thrice per week with calipers. Animals were removed from study and euthanized if any one tumor reached 4 cm in length or 4000 mm^3^, sum of tumor volumes exceeded 4000 mm^3^, > 15% weight loss, BCS < 3, or skin/tumor ulceration. Following euthanasia, animals underwent necropsy and select tissues were collected for subsequent analysis. Rats were euthanized by regulated flow CO_2_ asphyxiation followed by bi-lateral thoracotomy or under deep anesthesia with isoflurane and vital organs were removed.

### Mouse fibrosis study

Female 4- to 6-week-old CD-1 mice (Charles Rivers Labs) were randomized into 4 cohorts of 5 mice. Each mouse was implanted subcutaneously in the dorsal flank with a fulvestrant eluting implant cut to 1 cm in length. Cohorts of mice after 2, 4, 8, and 16 weeks were euthanized by CO_2_ asphyxiation followed by cervical dislocation. Tissue surrounding the implant was harvested, fixed in 10% neutral buffered formalin and paraffin embedded. Embedded tissue was sectioned (5 µM) perpendicular to the implant and trichrome stained to visualize fibrotic capsular tissue. Stained tissue sections were imaged with a Keyence BZ-X800E microscope (Itasca, IL). Slides were scanned and images captured with a 4 × lens and color camera. The BZ-X800 analyzer was used to stitch tiled images to reconstruct tissue sections and measure capsule thickness. Fibrotic capsule measurements were taken at ~ 200 µm intervals along the circumference of the implant, where possible, ≥ 50 per time point.

### Sheep studies

Adult female Suffolk Cross sheep (N = 2), 180 to 350 pounds, were used to assess safety and fulvestrant tissue distribution from fulvestrant eluting implants. A single implant was surgically placed between the abdominal wall and mammary tissue at the base of the udder, such that the implant lay flat against the abdominal wall. Implants were oriented with spiraled tubing facing mammary tissue. After approximately 30 days post-implantation, animals were euthanized, necropsied, and select tissues were collected and flash frozen for bioanalysis. The udder and tissue surrounding the implant were fixed in 10% neutral buffered formalin for histopathological analysis.

### Animal usage guidelines

The rat, mouse, and sheep studies were conducted in accordance with guidelines and regulations set forth in UCSF Laboratory Animal Resource Center approved protocols: AN180895 (Rat and mouse studies) and AN191728 (Sheep study).

### Tissue bioanalysis

Select rat and sheep solid tissues were homogenized (Tissuelyser II, Qiagen), spiked with the internal standard carbutamide, and fulvestrant and carbutamide were extracted by protein precipitation using acetonitrile. Extracted samples were analyzed using a Shimadzu Prominence HPLC coupled to a Sciex API-4000 Qtrap mass spectrometer (Applied Biosystems) with electrospray ionization in the positive ion mode. Samples were injected (2 µL) and separated using an ACE C_8_ column (Avantor) with an isocratic method (90% acetonitrile, 5 mM NH_4_Ac, 1% formic acid). Transitions monitored were 607.305 to 589.300 and 271.738 to 156.200 for fulvestrant and carbutamide, respectively. Fulvestrant standards from 0.5 to 2000 ng/mL, with a 1 ng/mL lower limit of quantitation, were used to estimate fulvestrant sample concentrations.

For plasma sample quantitation, fulvestrant was extracted by liquid–liquid extraction using n-hexane–isopropanol, to which fulvestrant-d3 was spiked as internal standard. Samples were separated using a ACQUITY UPLC (Waters) coupled to a BEH C_18_ column with an acetonitrile gradient containing NH_4_Ac (5 mM). Fulvestrant and Fulvestrant-d3 transitions (− 605.2 to − 427.4 and − 608.2 to − 430.4, respectively) were monitored with a Sciex 6500 triple quadrapole mass spectrometer (Applied Biosystems) with electrospray ionization in negative mode. Fulvestrant standards from 0.025 to 10 ng/mL, with a 0.025 ng/mL lower limit of quantitation, were used to estimate fulvestrant sample concentrations.

### Histopathology

Formalin fixed sheep mammary tissue was paraffin embedded, sectioned (4 µm), and H&E stained by HistoTec Laboratory (Hayward, CA). Photomicrographs of stained tissue sections were acquired with a Nikon Eclipse Ci microscope with a 20 × lens and a SPOT camera. Individual images were stitched with SPOT 5.6 Image Capture software to recreate complete stained sections. Dr. Narayan R. Raju, DVM, MVS, PhD, DACVP (Pathology Research Laboratory, South San Francisco, CA) evaluated tissue histopathology.

#### Statistics

For the DMBA-induced breast cancer risk reduction study, tumor-free and overall survival distributions are presented as Kaplan–Meier curves and hazard ratio (HR) estimated by Log-rank. Animals were censored from analysis, only, if a fatality or required euthanasia occurred prior to initiation of systemic dosing. A tumor-free event was recorded if a mass was detectable by palpation in proximity to mammary tissue. Following dosing, a fatality for any reason was considered an event for both tumor-free and overall survival comparisons. For rat tissue fulvestrant levels, differences between two data sets were determined using a Mann–Whitney test, where a P-value ≤ 0.05 was considered statistically different. For comparison of multiple data sets, an ordinary one-way ANOVA followed by a multi-comparison analysis was conducted, with an adjusted *P*-value ≤ 0.05 considered statistically different. The curve fit of in vitro fulvestrant release was determined by a one phase exponential decay least-square fit. All statistical analysis was conducted using GraphPad Prism version 10.0.2.

## Results

### In vitro characterization of fulvestrant eluting implants

**Fig. 1:**
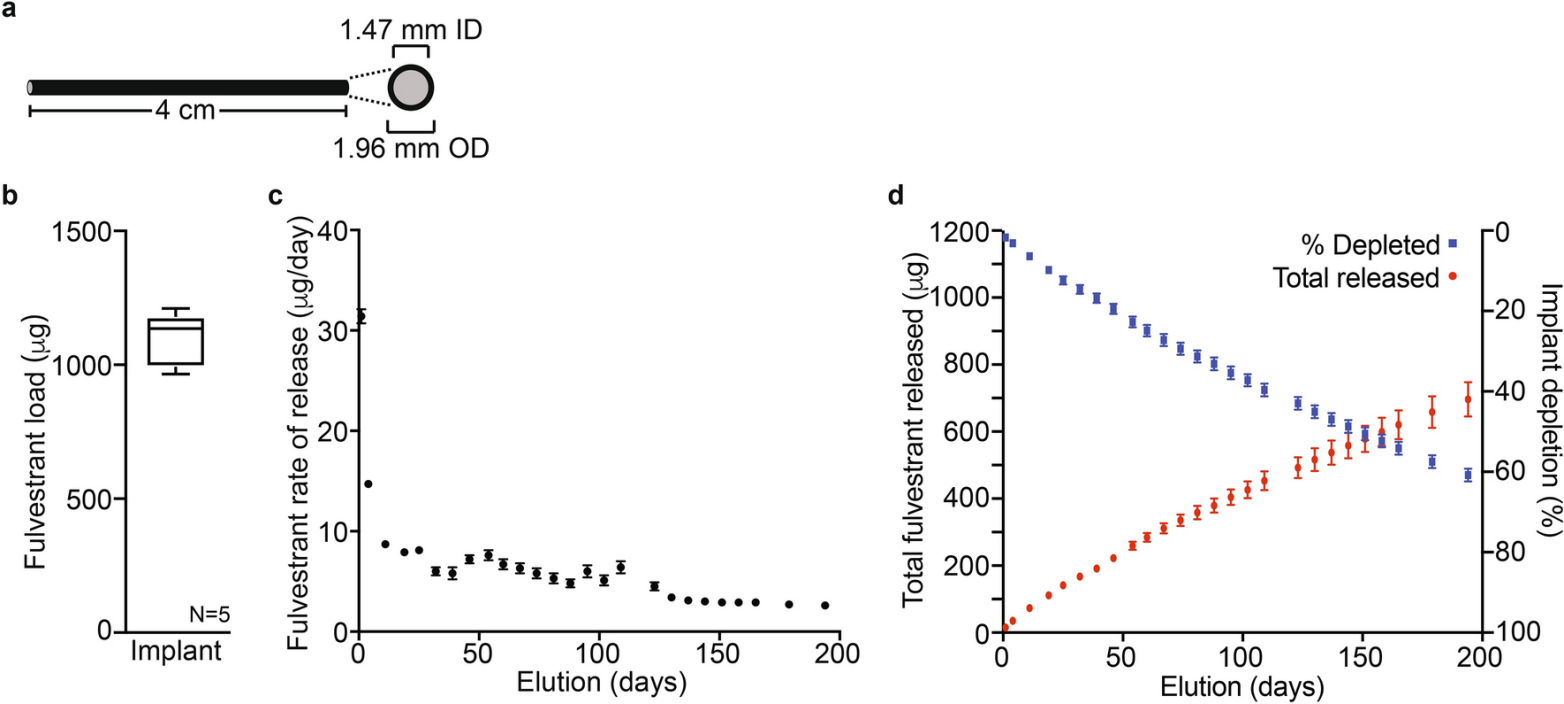
In vitro characterization of fulvestrant eluting implant. (**A**) Implants consisted of medical grade silastic tubing filled with MDX-4210 silicone elastomer containing 2.5% fulvestrant (w/w). (**B**) Fulvestrant content uniformity was assessed in formulated implants (N = 5) by tetrahydrofuran extraction and HPLC quantitation, presented in box and whisker graph. (**C**) In vitro elution of implants (N = 5) into 1% SDS normalized as a µg per day release rate is presented as the mean and standard deviation for each collection point. (**D**) The cumulative in vitro released fulvestrant (red, left Y-axis) and the estimated percentage of fulvestrant depleted from the implant (blue, right Y-axis) for each collection point is presented as the mean and standard deviation for each collection point.

Implants consisted of silastic silicone tubing (~ 0.25 mm wall thickness) and an inner core of fulvestrant (25 mg/g) embedded in cured silicone (~ 1.5 mm diameter) and cut to 4 cm in length (Fig. 1A). Solvent extraction demonstrated 4 cm length implant segments (N = 5) contained an average of 1096 ± 100 µg fulvestrant (Fig. 1B). An in vitro elution study was conducted to characterize the release profile of fulvestrant. Implants (4 cm, N = 5) were placed in vials and allowed to elute into a solution of 1% SDS. Implants were serially transferred to new vials with 1% SDS at the indicated times to ensure the elution rate was not diminished by the fulvestrant already eluted and solubilized. Fulvestrant elution from these implants was evaluated for 194 days. The elution rate profile exhibited 2-phase kinetics (Fig. 1C), which were characterized by an initial burst of release (~ 31 µg/day after 24 h) that rapidly decreased through day 11 (~ 9 µg/day), followed by a relatively slow decay to the end of the study (~ 3 µg/day). After collection of the final time point (day 194), remaining fulvestrant in each implant was extracted to determine percent depletion for each (Fig. 1D). The sum of total fulvestrant eluted and extracted was used to calculate total fulvestrant formulated in each implant. The calculated mass balance (1149 ± 113 µg/implant) was comparable to fulvestrant amounts determined from manufactured implants of the same batch (1096 ± 100 µg fulvestrant, Fig. 1B). Over the course of the study, the implants delivered 696 ± 51 µg of fulvestrant or 61.8 ± 1.6% of the total content (Fig. 1C).

### Locally delivered fulvestrant delays breast cancer in the 7,12-dimethylbenz[a]anthracene (DMBA)-treated female rat

DMBA-induced breast cancer in female Sprague–Dawley rats has been shown to closely recapitulate human estrogen receptor positive-hormonal therapy sensitive disease evolution. Following a single dose of DMBA, one or more tumors begin to arise within the mammary tissue typically between 100 and 200 days^34^. Systemic treatment with the anti-estrogen tamoxifen can prevent or inhibit growth of established tumors^35^. As such, this model was chosen to test the hypothesis that locally delivered fulvestrant from a drug eluting implant can be used to prevent or delay breast cancer.

**Fig. 2:**
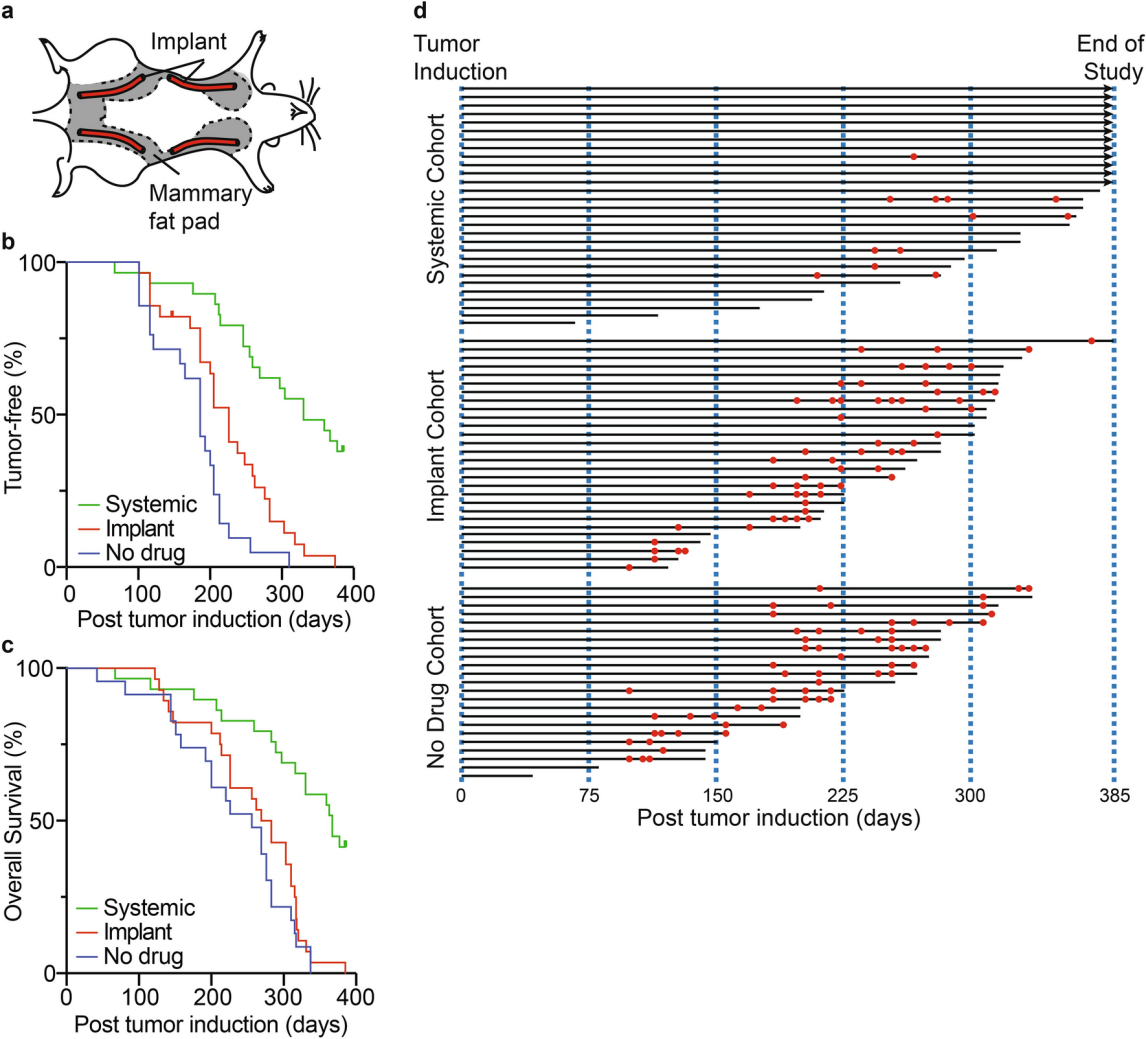
Efficacy of the fulvestrant-eluting implant compared to systemic fulvestrant and animals receiving no drug in a rat breast cancer prevention model. (**A**) Schematic of implant placement in rats. (**B**) Kaplan–Meier graph of tumor free animals receiving no treatment (blue), systemic fulvestrant (green), or locally delivered fulvestrant (red). (**C**) Kaplan–Meier graph of overall survival for animals receiving no treatment (blue), systemic fulvestrant (green), or locally delivered fulvestrant (red). (**D**) A swimmer’s plot of survival is presented for each animal in the three cohorts (black lines). Over-laid on the plot are times of tumor formation (red dots).

Mature female Sprague–Dawley rats (N = 90) were randomly divided into three cohorts: (1) no drug, (2) systemic fulvestrant, and (3) locally delivered fulvestrant. Of these, 10/90 animals died or required euthanasia within 7 days of DMBA administration. Remaining animals received either systemic fulvestrant treatment (N = 29), fulvestrant delivered locally via drug eluting implant (N = 28), or sham implants without fulvestrant (N = 23) (Fig. 2A). The primary endpoints of this study were time to first tumor occurrence and overall survival. The study was concluded when all animals in the locally treated cohort reached a study endpoint (Table 1). Similar to previous reports, animals receiving systemic fulvestrant exhibited significantly prolonged time to first tumor (median 330 days) compared to locally treated (median 226 days, HR = 0.308, *P* < 0.0001) and animals receiving no drug (median 186 days, HR = 0.222, *P* < 0.0001). Only 6 of 29 animals receiving systemic fulvestrant developed mammary tissue-associated tumors by the conclusion of the study (Fig. 2B, D). Implant-delivered fulvestrant also significantly delayed time to first tumor compared to animals receiving no drug (median 226 versus median 186 days, HR = 0.510, *P* < 0.010). Systemic administration of fulvestrant, further, increased animal overall survival (median 367 days) compared to both locally treated (median 276 days, HR = 0.299, *P* < 0.0001) and animals receiving no drug (median 256 days, HR = 0.259, *P* < 0.0001). Although locally treated animals exhibited numerically longer overall survival, this did not reach statistical significance (HR = 0.763, *P* < 0.304) (Fig. 2C). For both locally and systemically treated animals, no benefit was observed in extended survival following development of the first tumor (Fig. 2D).

**Table 1:** Rat breast cancer prevention study cause of death.

Cause of death	Treatment cohort *(N, %)*		
	Systemic (N = 29)	Local (N = 27)	Untreated (N = 23)
Tumor burden	1 (3.4)	19 (70.4)	19 (82.6)
End of study			
Non-tumor bearing	12 (41.4)	–	–
Tumor bearing	1 (3.4)	–	–
Ulcerated mass/skin^A^			
Non-tumor bearing	7 (24.1)	–	–
Tumor bearing	3 (10.3)	3 (11.1)	1 (4.3)
Weight loss/BCS^B^			
Non-tumor bearing	4 (13.8)	2 (7.4)	3 (13.0)
Tumor bearing	1 (3.4)	3 (11.1)	-

^A^Non mammary tissue associated lesions.

^B^Body condition score < 2.

Findings from our study demonstrate that the anti-estrogen fulvestrant significantly delays tumor formation and potentially prevents breast cancer, as well as increasing survival when provided systemically in the Sprague–Dawley rat DMBA model which served as a positive control. Here, we show for the first time that fulvestrant when provided locally via a drug eluting implant can also delay tumor formation.

### DMBA rat study fulvestrant tissue biodistribution

A key hypothetical benefit to local fulvestrant delivery as a means to prevent breast cancer is achieving efficacious target tissue drug levels, while substantially minimizing off target exposure. Plasma samples of rats in systemic and locally-delivered fulvestrant were collected on days 45, 72, 101, 130, 164, 172, and at time of necropsy for animals that exited the study. At the time of necropsy additional tissues were collected including mammary fat pads, kidneys, liver, and tumors. Additionally, all implants of the locally treated cohort were collected and the remaining fulvestrant in the implants was measured to calculate the cumulative release from the implant at the time of harvest.

**Fig. 3:**
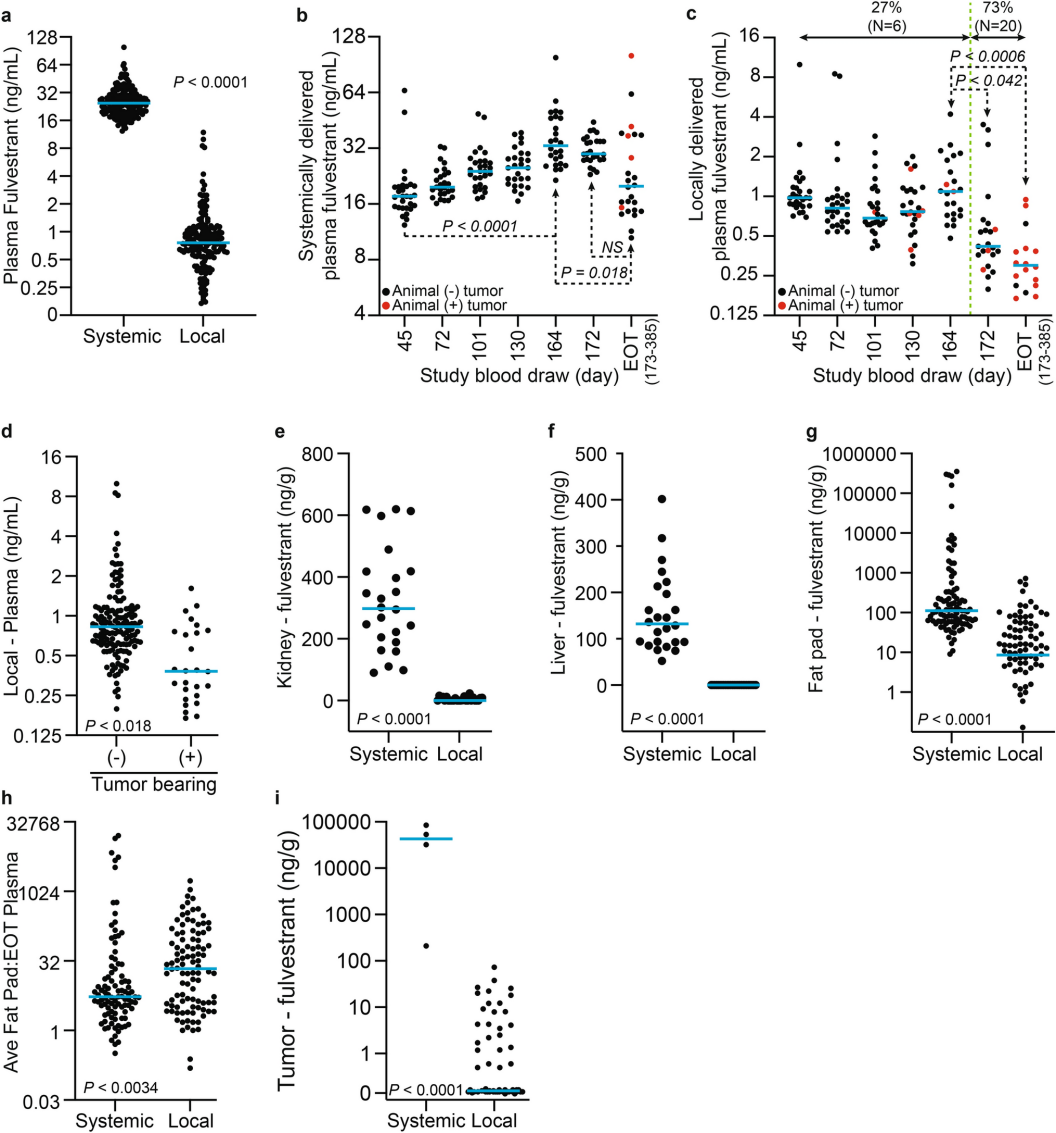
Fulvestrant biodistribution in the rat breast cancer prevention study. (**A**) Dot plot comparing the fulvestrant concentration in all plasma samples collected (in-life and end of study samples) in local versus systemic delivery. Dot plot of fulvestrant plasma concentration in animals receiving systemic (**B**) or locally (**C**) delivered fulvestrant. End of treatment (EOT) samples were collected between 173 and 385 days post tumor induction. Red dots indicate animals bearing tumors at the time of plasma collection. In (**C**), percentage and number of animals bearing tumors is presented up until day 164 and after through end of study (demarcated by green dashed line). (**D**) Dot plot of fulvestrant plasma concentration in locally treated animals comparing tumor ( +) versus non-tumor (-) bearing animals is presented. Dot plots of fulvestrant concentration in kidney (**E**), liver (**F**), mammary fat pad (**G**), and tumor (**I**) comparing local versus systemic delivery are presented. In (**I**), the asterisk indicates that fulvestrant was undetectable in 38 tumors arising in animals receiving locally delivered fulvestrant. (**H**) Dot plot of the mammary fat pad to end of study plasma fulvestrant ratio comparing local versus systemic delivery is presented. Blue lines indicate median values.

When samples across all times are compared, animals receiving systemic treatment exhibited ~ 32-fold higher median fulvestrant plasma concentrations compared to local treatment (24.65 versus 0.76 ng/mL, *P* < 0.0001) (Fig. 3A). Over the course of the study (Fig. 3B), the median plasma fulvestrant concentration in the systemic cohort increased through day 164, and then plateaued through day 172. Tumor occurrence and animal euthanasia started after day 173, which resulted in a considerable increase in plasma level variance and a decrease in median plasma levels. Yet there was no clear correlation between end of treatment (EOT) fulvestrant plasma levels and the presence of tumors or tumor burden. For local treatment (Fig. 3C), median fulvestrant plasma remained low and stable through day 164, after which it significantly dropped. For this cohort, 20 of 28 animals developed the first tumor following day 164. However, compared to the systemic cohort, across all samples, tumor bearing animals had significantly less median plasma fulvestrant levels (0.38 ng/mL) than non-tumor bearing animals (0.83 ng/mL, *P* < 0.018, Fig. 3D). In addition, of the 18 animals in this cohort for which plasma samples were collected, 17 had fulvestrant plasma levels below 1 ng/mL at the time of tumor development, suggesting tumor occurrence results from decreased fulvestrant in a specific implant or a decrease in fulvestrant release over time.

The kidney and liver are the primary organs of fulvestrant metabolism and clearance and exhibit highest tissue concentrations across animal models and humans^33^. In animals systemically treated, both kidney (298.1 ng/g, Fig. 3E) and liver (132.4 ng/g, Fig. 3F) exhibited greater median fulvestrant concentrations than found in plasma (24.65 ng/mL, Fig. 3A). In stark contrast, minimal to no fulvestrant was detected in the kidney (median 0.15 ng/g) and liver (undetectable) of locally treated animals. Mammary fat pads were divided by quadrant and individually assessed for fulvestrant concentration. Consistent with other tissues, median fulvestrant concentration in the mammary fat pads of the systemic cohort was significantly higher than that in the local cohort (111.3 versus 8.5 ng/g, *P* < 0.0001, Fig. 3G). However, the local cohort exhibited more efficient on target delivery with a ratio of median mammary fat pad to end of treatment plasma ratio of 21.8 versus 5.4 (*P* < 0.0034) for the systemic cohort (Fig. 3H). All tumors evaluated in the local treatment cohort (0 to 72.6 ng/g range, N = 62) had a lower fulvestrant level than those in the systemic treatment cohort (211 to 85,092 ng/g range, N = 4). Furthermore, 36 of the 62 tumors evaluated in the local cohort had undetectable fulvestrant (Fig. 3I) suggesting tumors arose due to limited fulvestrant delivery.

As hypothesized, local fulvestrant treatment resulted in significantly less systemic exposure. Furthermore, on target fulvestrant delivery was more efficient for local compared to systemic delivery. Tumors that arose in locally treated animals primarily occurred when fulvestrant delivery diminished, indicted by a significant drop in plasma levels over this span. Consistent with decreased delivery, these tumors were found to have minimal to no fulvestrant levels. In contrast, the few tumors that arose in systemically treated animals, which maintained relatively high plasma fulvestrant levels through end of treatment compared to locally treated animals, had substantially higher fulvestrant levels and thus are likely non-responsive to fulvestrant.

### Expected versus actual fulvestrant delivered via the drug eluting implants

**Fig. 4:**
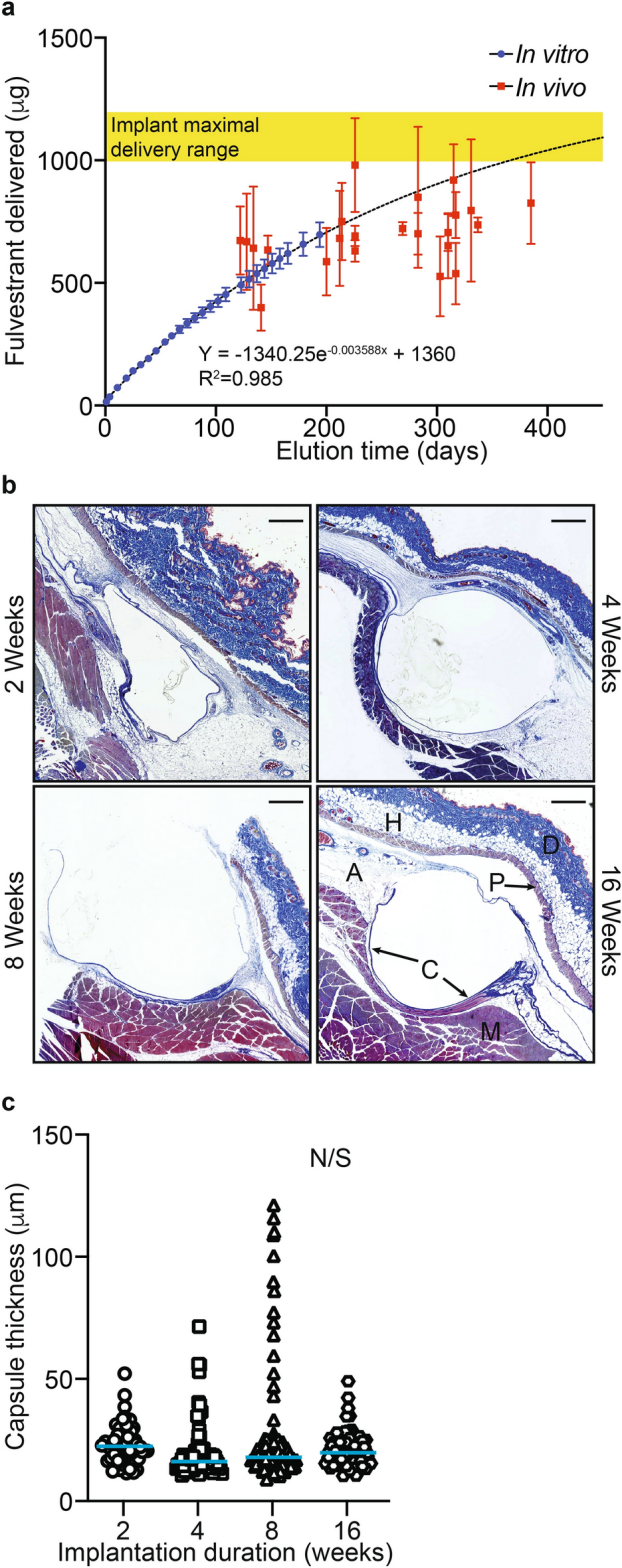
Expected versus actual fulvestrant delivery from implants in the breast cancer prevention study and the impact of fibrotic encapsulation. (**A**) Fulvestrant delivered versus elution time is plotted. In vitro elution data is plotted (blue) and a non-linear regression curve fit is presented and used to extrapolate elution beyond the ~ 200 days of collected data. Over laid (red) are fulvestrant delivered values determined from implants recovered from animals at the time of necropsy (mean of four implants with standard deviation per animal). Tissue surrounding scaled implants (1 cm length) subcutaneously placed in the dorsal flank of CD-1 female mice were collected at the indicated times (N = 5 per time point), formalin fixed, and trichrome stained to visualize the fibrotic capsule. Examples of stained cross-sections are presented (**B**) The dermis (D), hypodermis (H), panniculus carnosus (P), adventitia (A), skeletal muscle (M), and fibrotic capsule (C) are indicated in the 16-week image (Scale bar = 200 µm). (**C**) Comparative capsule thickness is presented. Median values indicated by blue bars.

EOT plasma samples from the locally treated animals were significantly diminished compared to earlier points in the study (Fig. 3C) and harvested tumors contained little to no detectable fulvestrant (Fig. 3G). This reduced delivery could be explained by a drop in the rate of release because of implant fulvestrant depletion, perturbation of release caused by fibrotic encapsulation of implants overtime, fulvestrant degradation, or potentially a combination of these. In vitro release rate characterization for 194 days (Fig. 1) did not result in the detection of novel fulvestrant degradation products or metabolites during HPLC quantitation that would suggest compound degradation over time (data not shown). From this idealized release experiment, designed to eliminate imperfect sink effects, a non-linear fit of the release data (R^2^ = 0.985, Fig. 4A) predicts ~ 79% and ~ 89% of implant fulvestrant was delivered by day 300 and 385, respectively. To assess actual delivery, retained fulvestrant was extracted from implants recovered during end of study animal necropsy. Substantial variance in extracted fulvestrant across the four implants from the same animal was observed. Implants from most animals (18 of 23) averaged delivery of fulvestrant near or less than the predicted amount based on extrapolation of the in vitro curve fit. Values exceeding the predicted release may be due to formulation variations or incomplete fulvestrant extraction resulting from implant adherent tissue. At necropsy, implants were observed to be associated with remnant fat or connective tissue to varying degrees, which may have affected extraction efficiency.

To better understand the potential influence of fibrotic encapsulation on implant fulvestrant elution, a subcutaneous mouse model was employed to assess encapsulation over time^36^. To this end, CD-1 female mice were implanted subcutaneously with 1 cm long versions of the fulvestrant eluting implant used in the rat breast cancer prevention study. Implanted mice were divided into four cohorts (N = 5 per cohort). Implants with surrounding tissue were harvested from one cohort after 2, 4, 8, and 16 weeks, respectively. The tissue was formalin fixed and paraffin embedded. Sections were trichrome stained to visualize fibrotic tissue and implant surrounding capsule thickness was measured (Fig. 4B, C). For individual implants, capsule development remained relatively consistent (ranging from 19.8 ± 11.3 to 24.5 ± 22.0 µm), punctuated by small regions of increased thickness. This was observed in the earliest cohort (week 2), however, no significant difference in capsule thickness was observed in subsequent cohorts through 16 weeks.

Taken together, these results suggest that implants delivered fulvestrant in vivo close to that predicted by the in vitro elution study. Less than predicted delivery was likely a combination of imperfect sink conditions surrounding implants and fibrotic encapsulation. However, the drop in plasma observed at later time points was more likely due to diminished fulvestrant implant reserves rather than decreased release from increasing encapsulation.

### Safety and tissue distribution of locally delivered fulvestrant in Suffolk cross ewes

**Fig. 5:**
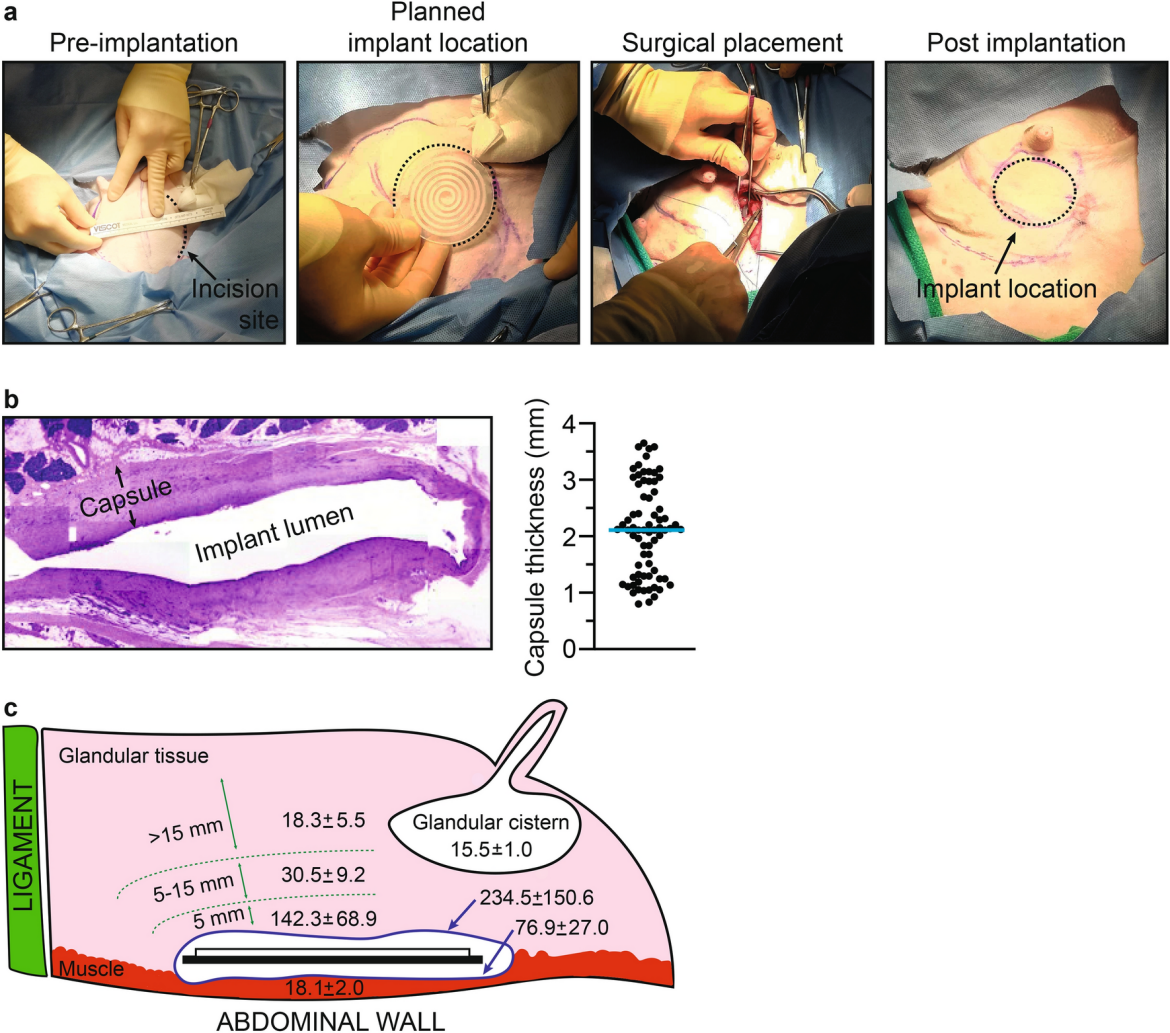
Evaluation of fulvestrant eluting implant in Suffolk Cross ewes. (**A**) Pictorial sequence of events during surgical placement of implant (left to right). (**B**) H&E stained cross-sectional image of fibrotic capsule and surrounding udder tissue (left) and dot plot of capsule thickness with blue bar indicating thickness median (right). (**C**) Schematic of ewe udder, implant position, and fulvestrant distribution relative to the suspensory ligament (green) and abdominal wall. Fulvestrant tissue concentration (ng/g) was evaluated in capsular tissue (blue), skeletal muscle adjacent to the implant (red), the glandular cistern, and glandular tissue adjacent to the implant (0–5 mm), near the implant (5–15 mm), and distal to the implant (> 15 mm).

Critical to efficacy of a drug eluting implant placed in the target tissue is sufficient distribution of the therapeutic throughout the mammary tissue. Ovine mammary tissue exhibits comparable histological features and volume to the human breast and^37,38^, as such, ewes have been used in a variety of procedure-based studies requiring a breast model^39–41^. To evaluate safety, target tissue distribution, and systemic exposure of fulvestrant delivered via a drug eluting implant, a Suffolk Cross ovine model was employed. Implants consisted of ~ 50 cm of silicone tubing filled with 2.5% fulvestrant cured in elastomeric silicone (w/w). The tubing was arranged in a spiral pattern and cured to a disc of silastic silicone (6 cm diameter, 0.3 cm thick) (Fig. 5A). In two ewes, this implant prototype was surgically placed within the udder and flat against the abdominal wall with the tubing face anteriorly directed into the glandular tissue. After 30 days, the animals were euthanized and underwent necropsy. Plasma, liver, kidney, and the implant containing udder were harvested for subsequent analysis. No gross abnormalities were observed by the study veterinarian.

Following implant removal, the harvested udder tissue was evaluated for histopathology. The implant lumens were found to be surrounded by a fibrous capsule (2.11 ± 0.81 mm, Fig. 5B), which multifocally extended into the interlobular tissue. Within the capsule, clusters of lymphocytes, neutrophils, and, to a lesser extent, plasma cells were observed. Further, pockets of macrophage and occasional giant cell infiltrate were found in the adipose tissue of the gland proximal to the implant. Beyond histological changes associated with mild fibrotic encapsulation, no microscopic lesions were found within the glandular tissue.

Systemic exposure resulting from locally delivered fulvestrant was assessed by quantifying drug in plasma and known tissue reservoirs, namely the kidney and liver, organs of metabolism and clearance (Table 2). Plasma levels were 1.2 ± 0.5 ng/mL, with a ~ threefold accumulation in liver and kidney tissue (3.3 ± 0.08 and 3.8 ± 0.81 ng/g, respectively). To assess fulvestrant distribution through the udder, 5 mm punch biopsies were taken from the capsule, muscle/fascia between the capsule and abdominal wall, glandular tissue within 0–5, 5–15, and > 15 mm from the capsule, and the glandular cistern (Fig. 5C). Highest fulvestrant values were found in the tubing-facing capsule (234.5 ± 150.6 ng/g) and glandular tissue adjacent to the capsule (142.3 ± 68.9 ng/g). Fulvestrant levels decreased with distance from the capsule. Fulvestrant levels were also detected in the glandular cistern (15.5 ± 1.0 ng/g) and tissues between the dorsal facing capsule and abdominal wall (18.1 ± 2.0 ng/g), however, as anticipated from the increased thickness of the backwall of the implant, these levels were lower.

**Table 2:** Sheep fulvestrant tissue biodistribution.

Tissue	Fulvestrant, ng/g	
	Mean	SD
Plasma^A^	1.2	0.5
Liver	3.3	0.8
Kidney	3.8	1.0
Udder		
Capsule		
Dorsal facing	76.9	27.0
Ventral facing	234.5	150.6
Glandular^B^		
0–5 mm	142.3	68.9
5–15 mm	30.5	9.2
> 15 mm	18.3	5.5
Muscle	18.1	2.0
Glandular cistern	15.5	1.0

^A^ng/mL.

^B^Distance from the capsule.

In summary, this 30-day sheep study demonstrated indwelling fulvestrant eluting implants exhibited no signs of acute local toxicity. Implants did provoke a foreign body reaction with moderate inflammation that did not prevent fulvestrant elution. Eluted fulvestrant penetrated through the fibrotic capsule and deep into the surrounding glandular tissue. In contrast, systemic exposure was low with plasma levels significantly lower than target tissue levels.

## Discussion

Individuals at high risk for developing breast cancer are afforded limited options for prevention. Both the surgical removal of all breast tissue or prolonged anti-estrogen therapy pose an undue physical and emotional toll and many women delay or forgo these interventions. Thus, we developed a long-term drug-eluting implant to administer an anti-estrogen solely to the breast to provide the benefits, but not the systemic side effects, of an oral anti-estrogen. To test this hypothesis, we assessed the ability of a fulvestrant eluting implant to prevent or delay breast cancer in the rat DMBA-induced breast cancer model^35^.

As previously shown in this rat model^42^, systemically delivered fulvestrant both effectively delayed tumor incidence and increased survival of animals. While not as strikingly as systemic delivery, local delivery via implants effectively delayed tumor onset in animals, however, this early implant prototype did not sufficiently impact survival. Detailed tumor biodistribution studies suggested that the implant containing 2.5% fulvestrant (w/w) delivered less drug than the systemic administration. Most tumors arose in animals receiving local fulvestrant when fulvestrant plasma levels dropped significantly suggesting depletion of drug in the implants. Once fulvestrant dropped below the effective therapeutic window shown in other preclinical models^43^, tumors rapidly formed. Furthermore, tumor fulvestrant levels were relatively low, significantly below levels known to inhibit tumor growth in other breast cancer models, with more than half having undetectable levels. Together, these findings suggest that an implant reformulated to deliver greater rates of fulvestrant for a longer duration could provide comparable risk reduction to systemic therapy. This and previous studies with silicone-based tubing implants demonstrate fulvestrant is released with zero order kinetics (DNS and Park *et al*^33^). Increasing the amount of fulvestrant in an implant extends the duration of release, while decreasing tubing wall thickness increases the release rate. Eliminating the wall and formulating fulvestrant homogenously in silicone elastomer significantly increases the release rate but alters the kinetics such that release exhibits exponential decay with diminished duration. New implant formulations evaluating these parameters are currently being developed to enhance drug release, while extending duration (DNS). 

Equal in importance to risk reduction for this approach is the significant improvement of on target versus off target fulvestrant delivery. Consistent with our previous mouse studies^33^, minimal off target fulvestrant tissue exposure was observed in rats and sheep receiving drug eluting implants. In addition, these animals exhibited a more favorable mammary tissue to plasma ratio than those receiving systemic dosing supporting our hypothesis that adipose rich mammary tissue would act to retain the eluted highly lipophilic fulvestrant. These effects were seen despite the fact that only a fraction of the implant drug eluting surface was in direct contact with the mammary fat pads in this rat model and not all of an animal’s fat pads were adjacent to a portion of the implant.

Implantable biomaterials often elicit a foreign body response from the host immune system resulting in fibrotic encapsulation. This response varies substantially depending on the biomaterial and geometry, its texture, anatomical location, implantation site trauma, and the individual^44–46^. Severe encapsulation and contracture are common complications associated with silicone breast implants often requiring a surgical intervention due to discomfort, pain, and/or for cosmetic reasons^47^. Our implants created minimal fibrosis in the studied models beyond two weeks. For indwelling drug eluting implants, a further concern is the impact of foreign body response on drug elution. Our data is supported by comparison to close surrogates to the fulvestrant eluting implants such as progestin eluting implants approved for contraception, namely Norplant™ and Jadelle™. Both of these silicone-based drug eluting implants are placed subcutaneously and provide sufficient drug for five years of effective birth control without serious complications or drug interference associated with encapsulation^48,49^. In this study, temporal assessment of the fulvestrant eluting implant in the subcutaneous dorsal flank of mice showed mild, but non-progressive encapsulation over four months, with capsule thickness comparable to previous rodent studies employing silicone implants^36,50^. The larger fulvestrant eluting implant form factor used in the sheep studies did produce a thicker capsule with moderate inflammation. However, this thicker capsule was comparable to that seen with silicone implants in other large animal studies^51–53^, and in women^53,54^. Yet, fulvestrant was found greater than 15 mm into glandular tissue from the implant capsule, consistent with progestin eluting implants whereby drug is able penetrate and diffuse across the fibrotic layer. When compared to transdermal delivery, an alternate approach of anti-estrogen delivery directly to the breast, the fulvestrant eluting implant compared favorably. Two neo-adjuvant phase II trials employing 4-hydroxytamoxifen gels have been conducted. In a study by Rouanet *et al*^55^, women with invasive breast cancer applied 4-hydroxytamoxifen gels of different concentrations (0.5 to 2 mg/day) daily to their breasts ranging from 2 to 3 weeks or received oral tamoxifen (20 mg/day). From collected tissues, 4-hydroxytamoxifen levels in both tumor (median 0.69–1.70 ng/g transdermal delivery versus 4.24 ng/g oral delivery) and non-malignant tissue (median 0.28–0.76 ng/g transdermal delivery versus 2.04 ng/g oral delivery) were quantified. In a similar study by Lee *et al*^29^, women with DCIS prior to surgery either applied 4-hydroxytamoxifen gel (2 mg/day per breast) or received oral tamoxifen (20 mg/day) for 6 to 10 weeks. Tamoxifen and its metabolites were measured in normal breast tissue adjacent to the lesion, with comparable z-4-hydroxytamoxifen levels in the oral (5.4 ± 2.8 ng/g) versus transdermal (5.8 ± 9.3 ng/g) cohorts. However, plasma levels were > fivefold lower in patients receiving transdermal therapy (0.2 versus 1.1 ng/mL). Furthermore, both studies found transdermal delivery significantly decreased tumor proliferation as measured by Ki-67 positivity. Due to the relative potency of fulvestrant versus 4-hydroxytamoxifen, finding 18.3 ± 5.5 ng/g beyond 15 mm from the implant in sheep suggests a therapeutically relevant dose is being achieved through this tissue within 1 month with low plasma levels. Moreover, a re-formulated drug eluting implant has the potential to deliver significantly greater levels of fulvestrant, while it is unclear whether greater delivery can be achieved with a transdermal application.

This study is limited by several weaknesses. The implant used in the rat breast cancer risk reduction study was formulated with a low percentage of fulvestrant (~ 2.5% w/w) due to manufacturing constraints. This is likely the reason for decreased drug measured in plasma and the formation of tumors in these animals and reduced efficacy compared to systemically treated animals. Newer manufacturing methods are capable of producing silicone formulations with drug as high as 70% (w/w). This study was further weakened by the number of ewe’s and time points evaluated to assess both safety and fulvestrant distribution through breast tissue. For this initial pilot study, however, a limited number of ewe’s and timepoints were selected to minimize the number of animals required in evaluating the early prototype before proceeding with further formulation optimization and large animal studies. Greater tissue sampling throughout the udder in subsequent studies would enhance the understanding of distribution. Finally, although gross pathology of organs and udder histopathology did not find significant tissue abnormalities, a more thorough evaluation of changes in breast tissue, specifically involvement of immune response and inflammation, as well as histopathology of major organs, is required to fully assess the safety and toxicity of this and subsequent implants.

In summary, this proof of principle study in the canonical hormone sensitive breast cancer rat model supports local delivery of fulvestrant via a silicone-based implant as a means to decrease the risk of breast cancer. Safety and biodistribution evaluation in the udder of ewes demonstrates indwelling fulvestrant eluting implants do not elicit significant toxicity and provide levels of a potent anti-estrogen deep into breast tissue comparable to oral delivery in women. Effort is currently being directed to improve formulations to maximize fulvestrant delivery and duration. Combined with these formulation refinements, subsequent large animal studies will aim to explore temporal relationships between release, breast tissue distribution, systemic exposure, and a more comprehensive evaluation of safety. These next step studies will position this therapeutic approach for translation into the clinic for further evaluation.

## Data Availability

The data sets used and/or analyzed during the current study are available from the corresponding author on reasonable request.
